# Supplementary material for: Does multifamily therapy help parents of adolescents with anxiety-based school refusal? A qualitative approach
Source: PLoS One. 2025 Oct 8;20(10):e0334037. doi: 10.1371/journal.pone.0334037 (PMC12507313; doi:10.1371/journal.pone.0334037)
Supplement: S1 File — (DOCX) [file pone.0334037.s001.docx]

**Parent interview guide**

As a participant in the MULTIFAST group, we are going to look at your experience in this group.

- Did you have any expectations of multi-family therapy before you started? What expectations? In what way did the multifamily therapy meet your expectations, or not, or only partially?

- Did you find any of the proposed activities interesting?

Did you find any of the proposed activities uninteresting or inappropriate?

- Did you experience any memorable moments during these sessions? Can you tell me about them?

Can you tell me about your emotions during the sessions you mentioned?

- Multifamily therapy offers several group formats: large groups, groups between fathers or mothers, as a family, with other parents, adolescents, etc. Where did you feel most comfortable? Which did you find most helpful? The most difficult?

- What did you think of the sessions where siblings were present? Did you find them different from other sessions? In what way?

- Would you suggest any changes for future groups?

What kind of changes?

- Could you describe how (adolescent's first name) is doing at the moment?

- What is (first name) doing at the moment? (Specify the teenager's current situation: schooling (what type of school, training, employment, project)

-Have you noticed any changes in (first name) between now and (give month before multifamily therapy began)?

- How are things with your family at present? Have you noticed any changes in the family between now and (give month before start of multifamily therapy)? What kind of changes?
