# Supplementary material for: Does multifamily therapy help parents of adolescents with anxiety-based school refusal? A qualitative approach
Source: PLoS One. 2025 Oct 8;20(10):e0334037. doi: 10.1371/journal.pone.0334037 (PMC12507313; doi:10.1371/journal.pone.0334037)
Supplement: Attachment — Submitted filename: Response to reviewers.doc [file pone.0334037.s004.doc]

**Title:** **Does multifamily therapy help parents of adolescents with anxiety-based school refusal? A qualitative approach**

**Manuscript N° PONE-D-24-44513**

*Answers to reviewers’ comments with modifications we would like to make to the text.*

*The proposed modifications are highlighted in yellow in this document and in the revised version of the manuscript.*

**Reviewer #1**

We would like to thank Dr. Mariola Giménez-Miralles for her comments, which helped us improve our manuscript. We hope that the changes made in response to her comments will meet her expectations.

1. The manuscript presents a scientifically valid study with appropriate controls and sample sizes. The methodology is detailed and ensures replicability. However, it would benefit from further justification of the sample size (e.g., power analysis).

**Answer and proposed modifications:**

Thank you very much for this comment. We continued inclusions until data saturation, that is, until the analysis of new data provided no new results (Fusch, Ness, 2015), which is recommended for qualitative analysis of interviews. Power analyses are not conducted in qualitative research. We included every participant in the intervention who agreed to participate: 84%.

*References*

Fusch PI, Ness LR. Are we there yet? Data saturation in qualitative research. Qual Rep. 2015;20(9):1408.

2. The conclusions are generally supported by the data, though a more in-depth discussion of study limitations would strengthen the manuscript

**Answer and proposed modifications:**

We thank Dr. Giménez-Miralles for this important remark. We have rewritten and detailed the study limitations:

The principal limitation of this study is that the choice of a qualitative method, by definition, means that we cannot demonstrate the therapeutic benefit of multifamily therapy for adolescents with anxiety-based school refusal and their parents. To do so, we would need to add a quantitative approach with statistical analysis to the qualitative approach. The presence of other factors, such as treatment intensification, may influence the parents' experience. The aim of this study is to explore parents' experiences of multifamily therapy, but it cannot quantitatively demonstrate its therapeutic benefit in the management of anxiety-based school refusal in adolescents. In the future, we plan to conduct a quantitative study to assess the effectiveness of multifamily therapy for adolescents with anxiety-based school refusal and their parents. This quantitative study will complement the qualitative approach presented in this paper.

**Modifications included in the revised text:**

Limitations and Prospects:

Because we chose a purely qualitative method, we cannot demonstrate the therapeutic benefit of multifamily therapy (MFT) for adolescents with anxiety-based school refusal (ABSR) and their parents. For example, the presence of other factors, such as treatment intensification, may play a role in the experience of the parents we interviewed. This study thus successfully explores parents' experiences of MFT, but it cannot quantitatively demonstrate its therapeutic benefit in ABSR in adolescents. In the future, we plan to set up a quantitative study to assess the effectiveness of MFT for adolescents with ABSR and their parents. This quantitative study will complement the qualitative approach presented in this paper.

3. Including raw data or a more detailed breakdown of summary statistics would enhance transparency. The manuscript complies with the PLOS Data Policy, but the authors should ensure that all underlying data is deposited in a public repository. The Data Availability Statement should be clear and in line with PLOS requirements. If there are any privacy or third-party restrictions, these should be explicitly stated.

**Answer and proposed modifications:**

We have not detailed the statistical results due to the choice of the qualitative method, which implies that we have not carried out statistical tests, although this is one of our future projects.

To be clearer about the Data Availability Statement, all data underlying the findings described in their manuscript are fully available without any restrictions.

All relevant data are within the manuscript and its Supporting Information files, i.e. the transcripts of the parents' interviews, as well as the interview guides. There are not any privacy or third-party restrictions

4. The manuscript is well-written in standard English, but some technical terms could be clarified for a broader audience.

**Answer and proposed modifications:**

We agree that we haven't adequately clarified some technical terms. Thank you for your comment.

**Modifications included in the revised text:**

Initial sentence: “Our research shows that encounters with other parents and family systems help to restore parents’ self-image, thanks to the identification and counter-identification movements that unfold in MFT.”

Modified sentence: “Our research shows that encounters with other parents and family systems help to restore parents’ self-image. Parents recognize that they have something in common with other parents and feel part of the parent group, thanks to the identification and counter-identification movements, respectively, that unfold in MFT.”

5. Figures and tables are clear, though more detailed legends are recommended for better understanding

**Answer and proposed modifications:**

We have detailed the legend of Table 1.

**Modifications included in the revised text:**

Table 1: Participants’characteristics and duration of school refusal

6. The manuscript appears to comply with ethical standards, with appropriate mentions of ethical approval and conflict of interest. The authors should confirm that the study is not a dual publication.

**Answer and proposed modifications:**

We confirm that the study is not a dual publication

**Reviewer #2**

We would like to thank the reviewer for these comments. Your expertise on the subject is invaluable in helping us to improve the paper and to reflect on our future research.

1. I sincerely thank the editor for giving me the opportunity to review the valuable study titled Does multifamily therapy help parents of adolescents with anxiety-based school refusal? A qualitative approach. The study is valuable and promising, but it requires some modifications that will enhance the study.
   Abstract; Method: Please include details about the sample: Are the parents fathers or mothers? What is the mean age and standard deviation for both fathers and mothers?

**Answer and proposed modifications:**

We thank the reviewer for these comments, which helped us to improve our paper.

We have added in the abstract the number of fathers and mothers, as well as the mean age and standard deviation for fathers and mothers, which we have also added in the results.

**Modifications included in the revised text:**

*Abstract:* Thirty-one parents (out of 37, 84%) agreed to take part in the study, 19 mothers and 12 fathers. The mean age for fathers was 51.5 (SD= 6.45) and 48.7 (SD=4.77) for mothers.

*Results:* Thirty-seven parents were asked to participate in this study, and 31 agreed (84%). The 6 who refused (or did not respond) either pointed out their lack of time or gave no explanation. Of the 31 parents, 19 were mothers and 12 fathers. The mean age was 51.5 (SD= 6.45) for fathers and 48.7 (SD=4.77) for mothers.

2. The results indicate that the changes in parents align with the main themes of the study. However, questions arise: How can these changes be attributed specifically to the multifamily therapy program? Could these changes be due to other uncontrolled factors during the treatment process or merely coincidental? How can the findings be interpreted in light of these potential biases?

**Answer and proposed modifications:**

This is indeed a central point, and we will attempt to respond to this very important observation. In this paper, we have chosen a qualitative method that explores the experiences of parents who have participated in the multifamily therapy (MFT) for anxiety based school refusal (ABSR) program. The results are based on an analysis of the parents' discourse. Parents are positioned as experts, i.e. those best placed to describe their experiences.

This qualitative approach cannot demonstrate that these changes can be attributed specifically to the MFT program, as other factors, such as the intensification of care and the other care offered to the adolescent, can contribute to the changes. This is especially true if the adolescent's condition has improved, which can improve communication within the family.

Analysis of the interviews highlights what parents describe about their experience of the MULTI FAST program. In addition, for several outcomes, parents report that it is their contact with other families facing the same problem that helps them to, for example, to feel less guilty, less abnormal, and less alone. Moreover, meeting other teenagers who also face ABSR helps them to better understand what their teenager is going through.

Your comment seems essential to us, because it is very important to pursue research in this field in order to demonstrate the therapeutic benefit of MFT for ABSR. The qualitative approach alone cannot do this.

In the future, we plan to pursue research in this area using a quantitative method, which may enable us to demonstrate the value of MFT. The qualitative and quantitative approaches are complementary. The qualitative approach enables us to gain a better understanding of participants' experiences and to analyze the factors that help them and how they feel. This enables us to improve the content of each session.

Since MFT is not yet used for ABSR with teenagers and their families, the first stage of qualitative analysis of parents' feedback is useful, not only to us but to others considering introducing this type of therapy. The second step will be to set up another study for which statistical analysis is possible. Your comment encourages us in this direction. Thank you very much.

**Modifications included in the revised text:**

Limitations and Prospects:

Because we chose a purely qualitative method, we cannot demonstrate the therapeutic benefit of multifamily therapy for adolescents with anxiety-based school refusal and their parents. For example, the presence of other factors, such as treatment intensification, may play a role in the experience of the parents we interviewed. This study thus successfully explores parents' experiences of MFT, but it cannot quantitatively demonstrate its therapeutic benefit in ABSR in adolescents. In the future, we plan to set up a quantitative study to assess the effectiveness of MFT for adolescents with ABSR and their parents. This quantitative study will complement the qualitative approach presented in this paper.

3. It is recommended to include additional elements related to family functioning, family resilience, and family competence in this section (Working with the Family). These aspects are essential for a comprehensive understanding of the family’s role and its capacity to support adolescents. The inclusion of these elements can also strengthen the argument and provide a broader perspective on effective interventions.

**Answer and proposed modifications:**

We would like to thank the reviewer for giving us the opportunity to explore in greater depth the key concepts of family functioning, family resilience, and family competence.

Families faced with an adolescent's ABSR must deal with a very difficult situation: their adolescent has been unable to attend school, sometimes for several months. Improving family resilience, that is, its ability to deal with challenges and adversity (Sabah et al, 2023) is therefore essential. The results show that family resilience is enhanced by encounters with other families facing the same problem, and by inter-family support.

The importance of working on family communication is another key point of the MFT program: family communication involves sharing information, ideas, thoughts, and emotions among family members (Smith et al 2009; Sabah et al 2023). In the third theme to emerge from the phenomenological analysis of parent interviews, parents described new interactions with better communication and a stronger family group. Some of the activities proposed during MFT sessions are designed to work on family communication. For example, during certain MFT sessions, we ask families to describe recurring blockages or conflicts within the family, and then to reflect on strategies for reacting and managing them differently. This requires them to work on family functioning, which involves the family members' ability to collaborate (Sabah, 2023).

Finally, family emotional expressiveness refers to the prevailing mode of emotional expression within the family context, encompassing both nonverbal and verbal cues (Halberstadt et al., 1995). For parents, successfully expressing their emotions within the MFT program in front of their family, and also hearing their adolescent express his or her emotions, are key moments in the program.

The study by Sabah and Alduais demonstrates the mediating role of the parent-adolescent relationship between family emotional expressiveness and adolescent mental health (Sabah, Alduais, 2024). This important point is in line with the objectives of MFT: to work on the parent adolescent relationship to improve adolescent mental health, but also to work on parents' expression of positive emotions toward their adolescent.

These points show the relevance to our MFT program of Olson's Circumplex Model of Marital and Family Systems (Olson, 2000), which emphasizes three primary aspects within family systems: cohesion, flexibility, and communication. This work, which the reviewer helped us discover, enables us to link theory to practice.

**Modifications included in the revised text:**

**Discussion:**

**Impact on family interactions**

Families faced with an adolescent's ABSR must deal with a very difficult situation: their adolescent has been unable to attend school, sometimes for several months. Improving family resilience, that is, its ability to deal with challenges and adversity (Sabah et al, 2023) is therefore essential. The results show that family resilience was enhanced by encounters with other families facing the same problem, and by inter-family support.

The importance of working on family communication is another key point of the MFT program: family communication involves sharing information, ideas, thoughts, and emotions among family members (Smith et al 2009; Sabah et al 2023). In the third theme to emerge from the phenomenological analysis of parent interviews, parents described new interactions with a better communication and a stronger family group. Some of the activities proposed during MFT sessions are designed to work on family communication. For example, during certain MFT sessions, we ask families to describe recurring blockages or conflicts within the family, and then to reflect on strategies for reacting and managing them differently. This requires them to work on family functioning, which involves the family members' ability to collaborate (Sabah, 2023).

Finally family emotional expressiveness refers to the prevailing mode of emotional expression within the family context, encompassing both nonverbal and verbal cues (Halberstadt et al., 1995). For parents, successfully expressing their emotions within the MFT program in front of their family, and also hearing their adolescent express his or her emotions, were key moments in the program.

The study by Sabah and Alduais demonstrates the mediating role of the parent-adolescent relationship between family emotional expressiveness and adolescent mental health (Sabah, Alduais, 2024). This important point is in line with the objectives of MFT: to work on the parent adolescent relationship to improve adolescent mental health, but also to work on parents’ expression of positive emotions toward their adolescent.

These points show the particular relevance to our MFT program of Olson's Circumplex Model of Marital and Family Systems (Olson, 2000), which emphasizes three primary aspects within family systems: cohesion, flexibility, and communication.

*References*

Sabah A, Aljaberi MA, Hajji J, Fang CY, Lai YC, Lin CY. Family Communication as a Mediator between Family Resilience and Family Functioning under the Quarantine and COVID-19 Pandemic in Arabic Countries. Child Basel Switz. 27 oct 2023;10(11)

Smith KM, Freeman PA, Zabriskie RB. An Examination of Family Communication Within the Core and Balance Model of Family Leisure Functioning. Fam Relat. Fev 2009;58(1):79‑90

Halberstadt AG, Cassidy J, Stifter CA, Parke RD, Fox NA. Self-expressiveness within the family context: Psychometric support for a new measure. Psychol Assess. mars 1995;7(1):93‑103

Sabah A, Alduais A. Intersections of family expressiveness and adolescent mental health: exploring parent–adolescent relationships as a mediator. Ment Health Soc Incl. 20 sept 2024. <https://www.emerald.com/insight/content/doi/10.1108/MHSI-06-2024-0104/full/html>

Olson DH. Circumplex Model of Marital and Family Systems. J Fam Ther. may 2000;22(2):144‑67

4. My important question is how the extraneous factors that could affect the results were controlled, since the study did not undergo experimental control. What ensures that the results are not due to other factors unrelated to the therapeutic program used?

**Answer and proposed modifications:**

We hope that the details given above answer this important question. This first step in a qualitative approach describes only the parents' experience and aims to analyze their words as faithfully as possible.

This first stage does not allow us to prove that the results described by the parents (such as better communication) are not due to other factors. A second step, with a quantitative approach enabling statistical analysis, is needed to demonstrate the therapeutic benefit of this MFT program.

Your feedback will help us to think about methodology in this second stage of our research. Your help will be invaluable in considering the scales we might use, in particular the Perceived Collective Family Efficacy Scale. We look forward to continuing our discussions and to benefiting from your expertise.

5. Please include the theoretical and practical implications of the study.

**Answer and proposed modifications:**

We have included the theoretical and practical implications of the study.

**Modifications included in the revised text:**

**Theoretical implications**:

This study sheds light on the importance of including both family and group therapy to help adolescents with ABSR. MFT allows us to work on intra-family communication and the expression of emotions within the family, so that they can better understand ABSR and at the same time benefit from the help of the group, so that families can support each other as they face the same problem.

**Practical implications:**

It would be helpful to systematically offer families programs where they can meet other families facing the same problem. A partnership between schools and health care services is essential to provide the best possible support for these families.

**Reviewer #3**

We would like to thank the reviewer for these comments. Your expertise on school refusal was invaluable and helped us to clarify and refine our study. In addition, your comments on the construction of the paper enabled us to rewrite certain parts and better organize the presentation. We are grateful for the time you took to review our paper. Your feedback was extremely helpful. We really appreciate it.

1. Thank you for the opportunity to review this manuscript. School refusal is a condition that needs additional evidence based interventions and supports as current options are not effective for a sizeable number of adolescents. Parent perspective on family-based interventions are useful to consider given parents central role in school attendance.
   Introduction
   Paragraph 1. The definition of school refusal in the first paragraph was quite fuzzy with some concepts at odds with common definitions such as Berg’s definition that are detailed in the next paragraph. For example, common to definitions of school refusal is parent knowledge of school non-attendance (see Berg’s definition), which contrasts the authors view that sometimes parents aren’t aware. Similarly, school refusal involves parents trying to get their child to school. This point differentiates school refusal from withdrawal – please see the Heyne et al paper that you have cited. Lastly school refusal is characterized by an association between emotional distress such as anxiety and school non-attendance. The authors may wish to review the difference between school refusal behavior as defined by Kearney and school refusal as described in the Heyne et al paper that is cited. They are different concepts. I believe the authors are focusing on school refusal or are the authors describing school attendance problems in this paragraph? The sentence on the International Network for School Attendance seemed disconnected from the sentence before and after. Please integrate or remove this sentence.

**Answer and proposed modifications:**

We fully agree that we must continue to explore the various possibilities for taking care of these adolescents, and we thank you for your interest in our multifamily therapy program, as well as in our study exploring the parents' point of view.

Thank you for your comment, which has helped us to clarify our point, which was not sufficiently clear. You are correct that the terms used are important because they reflect different concepts: precision and rigor are essential.

At the beginning of the first paragraph, we were describing the different school attendance problems, not the different types of school refusal.

In this study we are focusing on school refusal which involves parent knowledge of school non-attendance, parents trying to get their child to school and an association between emotional distress and school non-attendance. The adolescents we meet in our department present school refusal.

We have removed the sentence on the International Network for School Attendance which was disconnected from the sentence before and after.

**Modifications included in the revised text:**

School refusal is a real public health issue in many countries. Absenteeism from school has repercussions on future educational and professional plans (Aucejo, Romano, 2016; Gershenson et al., 2017) and increases the child's risk of dropping out of school.

School refusal can involve a variety of situations (Heyne et al., 2019; Havik, Ingul, 2021; Kearney, Silverman, 1990). In this paper we focus on one type of school attendance problem: school refusal, which is what we encounter most frequently in our adolescent psychiatry department. Anxiety, excessive fearfulness, unhappiness, unexplained physical symptoms, depressive affect, and sleep problems are all described with school refusal (Heyne, 2022). Parents are aware of these children's inability to attend class and have tried several strategies to help them return to school (Kearney, Silverman, 1990).

~~The International Network for School Attendance was established in 2018, to reduce school attendance problems by compiling, generating, evaluating, and disseminating information, assessment, and intervention strategies (https://insa.network).~~

*References*

Heyne D, Gren-Landell M, Melvin G, Gentle-Genity C. Differentiation between school attendance problems: Why and how? Cogn. Behav Pr. 2019;26:8‑34

Havik T, Ingul JM. How to Understand School Refusal. Front Educ. 2021;6(715177)

Kearney CA, Silverman WK. A preliminary analysis of a functional model of assessment and treatment for school refusal behavior. Behav Modif. 1990;14:340‑66

Heyne D. Developmental Issues Associated with Adolescent School Refusal and Cognitive-Behavioral Therapy Manuals A Practitioner Review. Z Für Kinder- Jugendpsychiatrie Psychother. 2022;50(6):471‑94

2. Line 67. Please specify the criteria used in the Havik et al study as it is not the same as Berg’s. Moreover, I suggest that the authors should note the lack of rigorous epidemiological data on school refusal in this paragraph.

**Answer and proposed modifications:**

It is indeed important to specify the criteria used in the different studies. In the study by Havik et al., 3.6 % of the students reported “quite often” on at least one item included in the school refusal related reasons for school non-attendance. (How often have you been absent from school in last three months because: 1. you were afraid or worried about something at school? 2. you would have felt sad or sorry if you went to school? 3. you wanted to avoid unpleasant situations at school? 4. you were afraid of making a fool of yourself at school?). That study interviewed 5465 students from 11 to 15 years old. The criteria used by Havik et al in this study are therefore different from Berg's criteria defining school refusal.

**Modifications included in the revised text:**

Looking more specifically at adolescent studies, Havik et al. found that 3.6% of 11–15-year-olds reported “quite often” on at least one item included in the school refusal related reasons for school non-attendance in the three months before the interview (Havik et al., 2015). That study interviewed 5465 students aged from 11 to 15 years old. Their criteria are therefore different from Berg's criteria defining school refusal. In the community sample of 11–17-year-olds studied by Steinhausen et al., 6.9% reported fear of going to school (Steinhausen et al., 2008). No specific gender differences were found for the duration of school absence (Johnsen et al., 2022).

These data show the difficulty in obtaining rigorous epidemiological data on school refusal, as the criteria and the populations vary widely from one study to another.

*References*

Havik T, Bru E, Ertesvåg SK. Assessing reasons for school non-attendance. Scand J Educ Res. 2015;59:316‑36

Steinhausen HC, Müller N, Metzke CW. Frequency, stability and differentiation of selfreported school fear and truancy in a community sample. Child Adolesc Psychiatry Ment Health. 2008;2:17

Johnsen DB, Lomholt JJ, Heyne D, Jeppesen P, Jensen MB, Silverman WK, et al. Sociodemographic and clinical characteristics of youths and parents seeking psychological treatment for school attendance problems. PloS One. 2022;17(1):0261449

3. Line 73-77. I found these sentences to be a little speculative/colloquially written, particularly the phrase teenagers often hear. I think it is better to utilize the small amount of epidemiological data to demonstrate a higher prevalence of school refusal in adolescents compared to children, if this is demonstrated to be the case. I think this paragraph could be more focused on the subheading. For example, children who refuse school are also bullied.

**Answer and proposed modifications:**

Following your suggestion, we have refocused the subject on epidemiological data which demonstrate a higher prevalence of school refusal in adolescents compared to children.

The issue of bullying and its correlation with school refusal is essential.

**Modifications included in the revised text:**

~~First of all, adolescence is an age when peer relationships are very important for development. What's more, the pressure on adolescents to make career choices and achieve academic results is much greater than for younger children. Teenagers often hear the phrase: "What are you going to do with your life?" Unsurprisingly, then, they~~ Adolescents have a higher incidence of anxiety-based school refusal (ABSR) than children.

(…)

The issue of bullying must also be addressed. Indeed, in France, nearly half of parents whose teenager has ABSR report that their child has been bullied at school (Benoit et al, 2020). The correlation between bullying and school refusal must be considered in caring for both children and adolescents with school refusal.

*References*

Benoit L, Harf A, Moro MR. Phobie scolaire. Retrouver le plaisir d’apprendre. Vigot. 2020. (Solutions)

4. Line 97. This paragraph presents multiple ideas – that family know the child best and that family interventions work for adolescent conditions; the latter point does not necessarily make family involvement “essential”.

**Answer and proposed modifications:**

Thank you for your comment, we have refocused on more rigorous arguments about the value of involving families:

1. the effectiveness of systemic family therapy

2. the higher prevalence of mental health problems among parents of adolescents with school refusal

3. most psychosocial treatments for school refusal do include a parent component.
(we describe this part in detail in the following paragraph)

**Modifications included in the revised text:**

Working with the family

~~The role of the family is essential. Why?~~

~~The first reason is simply that family members are the ones who live with the adolescent, know him or her best, and will be involved. It is extremely difficult and ineffective to work with an adolescent without working with the family members who are present~~

A first reason to offer family interventions is that systemic family therapy has been shown to be effective in adolescents’ psychiatric disorders.

5. Line 115-116. I agree with the assertion that a significant proportion of teens don’t respond to current treatments however this statement needs a reference from the treatment literature (the Chockalingham reference isn’t about treatment but parenting factors associated with school refusal). I suggest the authors look at Brandy Maynard’s review of treatments which describes that most psychosocial treatments for school refusal do include a parent component.

**Answer and proposed modifications:**

Brandy Maynard’s review is a very important paper, which we found very helpful in writing our paper. Maynard’s review is referenced on line 113. We have outlined the key findings of this review.

**Modifications included in the revised text:**

Maynard’s review includes 8 studies, describing different treatments for school refusal among children and adolescents; 6 of them actively involved parents (Maynard et al., 2018).

*References*

Maynard BR, Heyne D, Brendel KE, Bulanda JJ, Thompson AM, Pigott TD. Treatment for school refusal among children and adolescents: A systematic review and meta-analysis. Res Soc Work Pract. 2018;28:56‑67

6. Line 153-157. It would be helpful to specify if the studies cited to support MFT were adult studies or adolescent studies. From reading the titles of the references it seems that at least several are. The level of evidence with adolescents seems worthy of comment in order to build an argument for the use of this approach for school refusal.

**Answer and proposed modifications:**

Thank you for your comment. We have now specified which articles investigating the effectiveness of MFT studied adolescents.

**Modifications included in the revised text:**

MFT subsequently developed all around the world, for multiple issues and disorders (Gelin et al., 2018).

~~It has proven to be effective in managing eating disorders, mood disorders, schizophrenia, and first psychotic episodes, addictions, autism spectrum disorders, attention deficit hyperactivity disorder, post-traumatic stress disorder, intrafamilial abuse, and borderline personality disorder~~

Among the studies showing the effectiveness of MFT, several include adolescents, in particular, for eating disorders (Baudinet et al., 2021), autism spectrum disorders (DaWalt et al., 2018), attention deficit hyperactivity disorder (Lo, Ma, 2023), conduct disorder (Pérez-García et al., 2020), post-traumatic stress disorder (Hoskins et al., 2024), internet addiction (Liu et al., 2015), and drug abuse (Liddle et al., 2001). In the 2023 systematic review and meta-analysis of MFT effectiveness by Van Es et al., 55% of the studies reviewed included children or adolescents (Van Es et al., 2023).

*References*

Gelin Z, Cook-Darzens S, Hendrick S. The evidence base for Multiple Family Therapy in psychiatric disorders : A review (part 1): Evidence base for psychiatric MFT. J Fam Ther. 2018;40(3):302 325

Baudinet J, Eisler I, Dawson L, Simic M, Schmidt U. Multi‐family therapy for eating disorders: A systematic scoping review of the quantitative and qualitative findings. Int J Eat Disord., 2021;54(12):2095‑120

DaWalt LS, Greenberg JS, Mailick MR. Transitioning Together: A Multi-family Group Psychoeducation Program for Adolescents with ASD and Their Parents. J Autism Dev Disord., 2018;48(1):251‑63

Lo JWK, Ma JLC. Use of nature in a multi‐family therapy for Chinese families of adolescents with attention deficit hyperactivity disorder: A photo‐elicitation study. J Fam Ther. 2023;45(1):65‑80

Pérez-García M, Sempere-Pérez J, Rodado-Martínez JV, López DP, Llor-Esteban B, Jiménez-Barbero JA. Effectiveness of multifamily therapy for adolescent disruptive behavior in a public institution: A randomized clinical trial. Child Youth Serv Rev. 2020;117:105289

Hoskins D, Hernández M, Pérez A, Spampinato L, Tahir P, Chang T. A novel multifamily treatment targeting symptoms related to Latinx caregiver deportation. Psychol Trauma Theory Res Pract Policy. 2024;16(Suppl 2):S409‑16

Liu QX, Fang XY, Yan N, Zhou ZK, Yuan XJ, Lan J, et al. Multi-family group therapy for adolescent internet addiction: exploring the underlying mechanisms. Addict Behav. 2015;42:1‑8

Liddle HA, Dakof GA, Parker K, Diamond GS, Barrett K, Tejeda M. Multidimensional family therapy for adolescent drug abuse: results of a randomized clinical trial. Am J Drug Alcohol Abuse. 2001;27:651‑88

Van Es CM, El Khoury B, Van Dis EAM, Te Brake H, Van Ee E, Boelen PA, et al. The effect of multiple family therapy on mental health problems and family functioning: A systematic review and meta‐analysis. Fam Process. 2023;62(2):499‑514

7. Line 180. It is stated that the duration of school refusal was between 2 weeks and 18 months. This detail might be better suited to the methods. Indeed, the entire description of the MULTI FAST program appears better suited to the methods.

**Answer and proposed modifications:**

Thank you for your comment. We agree that the description of the MULTI FAST program more appropriately belongs in the methods section.

**Modifications included in the revised text:**

We have moved the description of the MULTI FAST program to the beginning of the methods section.

8. Line 237. Participants met Berg’s criteria. How was this established? What were the selection criteria? What was the minimum number of days missed of school?

**Answer and proposed modifications:**

All adolescents to whom the MULTI FAST program is offered are outpatients in our child and adolescent psychiatry department. The psychiatrist verifies that the Berg criteria have been met and makes the DSM-5 diagnosis. If Berg’s criteria are met and if the adolescent has at least one DSM-5 anxiety disorder as primary diagnosis, the MULTI FAST program is offered to the adolescent and his or her family.

The selection criteria apply to the adolescent:

Age between 12 and 18 years old

Meets Berg’s criteria (determined by psychiatrist)

Diagnosed with at least one DSM-5 anxiety disorder

Period of school absenteeism ranging from 2 consecutive weeks through 18 months

In selection criteria, period of school absenteeism could vary from 2 weeks to 18 months. We note, however, that the minimum number of school days missed in our population was 92 days, so already quite a long time.

**Modifications included in the revised text:**

**Methods**

All included parents participated in a MULTI FAST group, i.e., an MFT group focused on ABSR. ~~All the parents included had an adolescent aged between 12 and 18 years who presented with this form of school refusal.~~ ~~, i.e., a period of school absenteeism ranging from 2 consecutive weeks through 18 months, and meeting Berg's diagnostic criteria. All adolescents had at least one DSM-5 anxiety disorder as their primary diagnosis.~~

Selection criteria were: adolescent aged between 12 and 18 years, whose psychiatrist determined that they met Berg's criteria for school refusal (Berg, 1992) and had at least one DSM-5 anxiety disorder as primary diagnosis, whose period of school absenteeism range from 2 consecutive weeks through 18 months, and who had attended mainstream schools and had not presented any learning difficulties before school refusal began.

**Results**

Thirty-seven parents were asked to participate in this study, and 31 agreed. The 6 who refused (or did not respond) either pointed out their lack of time or gave no explanation. Of the 31 parents, 19 were mothers and 12 fathers. In our population, the minimum number of school days missed in our study was 92 days.

*References*

Berg I. Absence from school and mental health. Br J Psychiatry. 1992;161:154‑66

9. Line 239. What are “normal grades” and how was this established?

**Answer and proposed modifications:**

This sentence does indeed need clarification. What we meant was that the grades of the adolescents included in the research were either around or above the mean for the class. This information came from the teenagers' school reports for the current and previous two school years (provided by the parents). None of the adolescents participating in the MULTI FAST program for school refusal had previous learning problems in school and their learning was proceeding without any difficulties before the onset of school refusal.

**Modifications included in the revised text:**

Before they began refusing to attend school, they had attended mainstream schools ~~with normal grades~~ and had not presented any learning difficulties before school refusal began.

10. Line 241-242. Of those who were offered participation, how many consented to participate?

**Answer and proposed modifications:**

This was specified at the beginning of the results section:

Thirty-seven parents were asked to participate in this study, and 31 agreed (84%). The 6 who refused (or did not respond) either pointed out their lack of time or gave no explanation.

11. Line 284. By including parent age, country of birth, profession, parent age and so on, I am concerned that someone who knew someone who participated in the study could be identified. Could some of these variables be removed to better protect participant anonymity. In addition, I think there is a typographical error for Father 12 – Cameroon, rather than Cameroun?

**Answer and proposed modifications:**

To better protect the anonymity of the participants, we have removed Table 1. Instead, we describe in general, and not specifically for each parent, characteristics such as: age, country of birth, marital status, socio-professional category, age of the adolescent with school refusal, duration of school refusal,

**Modifications included in the revised text:**

*Participant’s characteristics:*

- Of the 31 parents, 19 were mothers and 12 fathers

-Parents’age: from 41 to 65 years old. The mean age was 51.5 (SD= 6.45) for fathers and 48.7 (SD=4.77) for mothers

-Country of birth: 71% of parents were born in France

-19% of parents were divorced and 6% single or not living with a partner

-Of the 31 participating parents, 5 (16.5%) were teachers and 5 more hourly workers, 4 (13%) unemployed and 4 others sales workers, 3 (10%) artists and 3 business owners, 2 (6%) engineers and two nurses, and one retired, another a doctor, and yet another a civil servant.

-Adolescents were aged from 13 to 17 years old, 48% were girls, 52% boys

-Duration of school refusal was: 5 to 16 months. In our population, the minimum number of school days missed in our study was 92 days.

12. Line 289. While I am not an expert in IPA, I didn’t think the concept of saturation was used for this type of analysis. Moreover, a justification for using IPA would strengthen the Data Analysis section.

**Answer and proposed modifications:**

Thank you for drawing attention to the concept of saturation. In fact, it was not data saturation that determined the number of parents included, since the research was offered to all parents who participated in the MULTI FAST program during the inclusion period. Nonetheless, at the end of the interview analysis, we found that data saturation had been reached, insofar as data analysis became redundant and no longer provided new elements. We consider this relevant given that in qualitative analysis more generally, reaching data saturation is considered a criterion for validity.

We have also rewritten the Data Analysis section to better justify and explain the IPA method, as we had not sufficiently specified the choice and content of this analysis method.

**Modifications included in the revised text:**

Data analysis

A phenomenological research design was employed to explore the experiences of parents who participated in our MFT groups for school refusal. Phenomenology is a nonprescriptive approach to research that allows the essence of experience to emerge, yet anchors data analysis in the participants’ unique representations (Patton, 2002). The aim is to explore personal experience and the subjective perception of an object or event. Our research approach is phenomenological in that it involves detailed examination of the participants’ personal perceptions and lived experiences. To analyze our interview data, we used the Interpretative Phenomenological Analysis (IPA) method (Eatough, Smith, 2008; Smith, Osborn, 2008), an established qualitative methodology used to explore in depth how individuals perceive particular situations they are facing and how they make sense of their personal and social world. Applying IPA, we conducted an in-depth qualitative analysis, beginning with a detailed case-by-case study of each interview transcript by an iterative inductive process. Specifically, to obtain a holistic perspective, we conducted several close detailed readings of each interview, noting points of interest and significance. Step by step, we proceeded to describe the analytic themes and their interconnections, while taking care to preserve a link back to the original account. IPA thus involves navigating between different levels of interpretation (Eatough, Smith, 2008). The last stage involves the production of a coherent ordered table of the themes (Smith, Osborn, 2008). The procedure for data analysis was inductive; data from the literature were analyzed secondarily. The data collection and analysis took place at the same time. In this type of study, sampling, continued data collection, and the development of themes and sub themes are interdependent and simultaneous. The analysis influences the data collection by leading us to redefine the research question, to find counter-examples, and to investigate new pathways (Mays, Pope, 1995).

Validity

To insure the validity of our qualitative research, we compared the researchers’ coding. Two trained researchers (CS, AH) independently coded and interpreted all of the parent interview data.

The two coders discussed the emerging codes in repeated meetings with other members of the research team (EH, SG, SM, ML, CH, CB), all of whom had read the transcripts. All the researchers are women except SM who is a man.

These discussions helped to identify potential themes in the data that might not yet have been captured by the codes and enabled us to clarify or modify the coding to increase the consistency and coherence of the analysis by ensuring that the themes we identified reflected the data accurately and that the analysis was not confined to one perspective. Multiple discussions allowed us to eliminate systematic differences due to variations in interpretation. Validity was also enhanced by the care we took to distinguish clearly between what respondents said and how we interpreted it or took it into account (Smith, Osborn, 2008). Member-checking (also known as informant feedback or respondent validation) was practiced, for it is a vital way for interpretive researchers to verify the trustworthiness of their research (Baxter, Babbie, 2004). When the qualitative analysis of the parents’ data was completed, a summary of the thematic results was mailed to the parents, who were asked to provide feedback, reactions, and comments. Participants were also asked to share these preliminary results with their partners who had been unable to take part in the interview. Eighteen of the 31 parents provided written or verbal feedback, which was incorporated in the final results. This methodological aspect of the study provided a source of testimonial validity for the qualitative results; it also enabled us to take participant feedback about their interpretation into account (Plummer, 2001) and to assess the degree to which the themes resonated with the parents’ experiences.

Results

Analysis of the 31 parent interviews revealed four main themes. Table 1 presents the main themes and subthemes that emerged from the qualitative analysis of the interviews. Data saturation was reached with the analysis of these 31 interviews, that is, the point at which in-depth analysis of the interviews no longer results in the emergence of new themes (Fusch et al., 2015). Data analysis becomes redundant and no longer provides new elements.

*References*

Patton MQ. Qualitative research and evaluation methods. 3rd éd. Thousand Oaks, CA: Sage Publications; 2002

Eatough V, Smith JA. Interpretative Phenomenological Analysis. Willing C, Staiton-Rogers W, editors. London: Sage Publications; 2008

Smith JA, Osborn M. Interpretative Phenomenological Analysis. Smith JA, éditeur. London: Sage Publications; 2008

Mays N, Pope C. Rigour and qualitative research. BMJ. 1995;311(6997):109‑12

Baxter LA, Babbie E. The basics of communication research. Belmont,CA: Wadsworth. 2004

Plummer K. Documents of Life 2. Sage. London; 2001

Fusch P, Ness L. Are We There Yet? Data Saturation in Qualitative Research. Qual Rep. 8 sept 2015: https://nsuworks.nova.edu/tqr/vol20/iss9/3/

13. Line 328-329. This quote didn’t seem to fit as well with the theme of a valued teenager.

**Answer and proposed modifications:**

We agree and have removed it for that reason.

**Modifications included in the revised text:**

*~~Q6 : Mother 17: "... he's able to rediscover that, rediscover his sense of humor ... it feels good. Yes, he's back.”~~*

14. The aim of the study was to explore the experiences of parents who participated in the MFT with the findings having implications for the therapy. From this description, I was expecting the findings to very much focus on the parent’s experience in the therapy, however, the findings seem to go much broader than this and delve into issues like parents experience of school. I understand that this is what came up in the therapy, however it seems to have implications beyond just the therapy which I think is an additional strength rather than an issue. Hence, I wondered if the goals of the study might similarly be broadened to help guide the reader.

**Answer and proposed modifications:**

Thank you for your comment, which is very much in line with what we found from the analysis of the parents' interviews. This study's aim was to explore the experiences of parents who had taken part in the MULTI FAST program. It was relevant because this was a new program offered in our department and because MFT for school refusal had not, to our knowledge, been described in the literature at the time we designed the study. It was therefore a first exploratory step of the research.

Nevertheless, we fully agree with you: what emerged from the analysis of the parent interviews is broader. For example, we did not expect parents to talk about their own experiences of school, and this subsequently helped us to focus certain sessions even more on the representations of school not only of the adolescents but also their parents. When parents' experience of school is very negative or even hostile, it is much more complicated for them to support their teenager towards a return to school, and they find it more difficult to establish a positive partnership with the school. We therefore work specifically on changing parents' perceptions of school.

**Modifications included in the revised text:**

Objectives

The aim of this paper, after this brief review of school refusal and MFT, is to explore the experiences of parents who participated in one of our MFT groups for ABSR so that we could examine their experiences and thereby improve the therapy we offer for the adolescents and families we treat. The qualitative method, which relies on analyzing the discourse of the people involved, putting them in the position of experts, is particularly well-suited to this context and this goal. More specifically, then, we seek to analyze the participants' discourse and thereby gain better insight into the processes leading to change and clinical improvement for patients and their families. In particular, analysis of these interviews has already enabled us to develop new objectives to be worked on during MFT sessions.

15. At times, I found the description of the results to be quite brief, and I noted that there was no description of any theme. Some subthemes are very briefly described, for example, School refusal and parent distress’ is described in less than two lines (Line 384-385).

**Answer and proposed modifications:**

We were faced with the following problem: the analyses of the parents' interviews were very rich, and it was difficult not to detail everything. We chose to list the sub-themes and illustrate them with quotations, to keep as close as possible to what the parents had to say. The sub-themes were grouped within the main themes, following discussions between members of the research team at the time of the transversal analysis of all the interviews.

We plan to write further papers focusing on specific themes, which will provide much more detail of the subthemes.

The subtheme of school refusal and parent distress has been described in greater detail.

**Modifications included in the revised text:**

Parents with children distressed by school refusal felt depressed (Q1), powerless (Q2), and pressured greatly by their child's school and the environment (Q3). They felt judged by those around them and unsure of how to react; they experienced moments of sadness and despair.

16. Line 450. What is the “timeline” activity?

**Answer and proposed modifications:**

The timeline activity is offered to families during one of the MFT program sessions. Each family is asked to draw an arrow representing a timeline. On this timeline, the family will draw the important events in their life. All family members present must first discuss and agree on which events they will choose to write on the timeline, and on what date they will choose to start the timeline.

In the second stage, there's time for discussion with another family and then in subgroups, with questions such as: “How does what the family has experienced influence the way you react to the school refusal?” or “Were you surprised by what the other family members chose to include as important events?” etc.

This activity thus has two parts: the first where each family draws its timeline, then the second which is a discussion between families and then in sub-groups around questions linked to the activity.

**Modifications included in the revised text:**

*Mother 3 about the timeline activity (*which requires the family to jointly place the important events in their life along an arrow representing a timeline)*: “It was quite joyful. It was nice to remember it all together. I thought it was really nice. We were laughing”*

17. Line 457. A description of the methods used to receive participant feedback are needed in the methods section.

**Answer and proposed modifications:**

We have detailed participant feedback in the Data Analysis section.

**Modifications included in the revised text:**

Data Analysis

Validity

Member-checking (also known as informant feedback or respondent validation) was practiced, for it is a vital way for interpretive researchers to verify the trustworthiness of their research (Baxter, Babbie, 2004). When the qualitative analysis of the parents’ data was completed, a summary of the thematic results was mailed to the parents, who were asked to provide feedback, reactions, and comments. Participants were also asked to share these preliminary results with their partners who had been unable to take part in the interview. Eighteen of the 31 parents provided written or verbal feedback, which was incorporated in the final results. This methodological aspect of the study provided a source of testimonial validity for the qualitative results; it also enabled us to take participant feedback about their interpretation into account (Plummer, 2001) and to assess the degree to which the themes resonated with the parents’ experiences.

*References*

Baxter LA, Babbie E. The basics of communication research. Belmont,CA: Wadsworth. 2004

Plummer K. Documents of Life 2. Sage. London; 2001

18. The start of the discussion could be strengthened by providing an overview of the study aims to re-orient the reader and provide a brief summary of the findings.

**Answer and proposed modifications:**

We thank you for this comment which helps us to make our paper clearer by starting the discussion with a brief summary of the findings.

**Modifications included in the revised text:**

Discussion

The aim of this study was to describe the experiences of parents who participated in an MFT program about their adolescent’s school refusal. The phenomenological qualitative analysis of the 31 interviews highlighted several results. The first was the importance of the group: parents emphasized that being with other families facing the same problem as they did —their adolescent's school refusal — made them feel less alone, less stigmatized, less judged, and less guilty. It thus enabled them to regain confidence in themselves and their own abilities. The results also showed that a child's school refusal led to a deep sense of parental distress, sadness, and helplessness. The MFT program allowed them to express this to their teenager and the rest of the family. Some parents discovered during the MFT program that they had a negative vision of school, i.e., they realized that as children or teenagers their school experience had been difficult and that this had affected how they talk about school with their teenager. Adolescents also discovered how their parents felt at school when they were pupils and were sometimes surprised by what they saw.

Another important result was the change in how parents looked at their teens. Rather than seeing them as failures, they realized in the MFT group that their children have important skills and can and do analyze what's going on for themselves and find solutions. They also understood better what their teenager needs, and how their reaction as parents can help or, on the contrary, increase the teen's anxiety.

Finally, parents described how participation in a MULTI FAST group led to changes in family interactions and communication and strengthened family bonds.

19. The discussion seemed a little disjointed at times and an extension of the results rather than a discussion of them. The concept of self-efficacy is raised in the discussion, but did not overtly feature in the results section. This is followed by a long description of the findings of the Carless et al paper which needed to be better integrated. Next the discussion focused on parental anxiety which again was not an overt theme in the results.

**Answer and proposed modifications:**

We have modified this part of the discussion so that it is more in line with the results, particularly for the concept of self-efficacy.

We have also rewritten the description of the findings by Carless et al. to integrate it better into the discussion.

The part of the discussion focused on parental anxiety was not relevant, as parental anxiety was not a main theme in the results. We have thus deleted it.

**Modifications included in the revised text:**

Improving parental self-efficacy

The results of the parent interview analyses reveal a high level of parental distress. As we saw in the interviews, parents described feeling judged, guilty, and powerless to help their teenager. The results also show that the MFT program enabled parents to regain their self-confidence and feel less guilty. They once again felt able to help their teenager without putting pressure on him/her, and to be part of the solution. Carless et al. studied ~~family functioning among adolescents with ABSR. The results showed that these refusals significantly affected family interactions, impairing communication, reducing both emotional expression and problem-solving skills, and worsening the poor role differentiation within the family. Parents were significantly more anxious and depressed than those in the general population. They also studied~~ the level of parental self-efficacy in ABSR situations and showed that the feeling of parental competence is an important prognostic factor (Carless et al., 2015) ~~one that may be diminished when parents have anxious or depressive disorders~~. One of the aims of the MULTI FAST program ~~treating this form of school refusal among adolescents~~ is to increase parents' sense of competence and self-efficacy in parenting.

~~Parental anxiety disorders may be present before the onset of school refusal, but they are also fostered by a sense of powerlessness in facing it. In any case, if the parents' anxiety level is high, they will be less able to help their teenager try out new strategies or change behaviors, because they will be too anxious themselves. So we must care for the parents too!~~

*References*

Carless B, Melvin GA, Tonge BJ, Newman LK. The role of parental self- efficacy in adolescent school-refusal. J Fam Psychol Am Psychol Assoc Div. 2015;29(2):162‑70

20. Orienting the reader to the capacity of MFT to improve mentalization in the introduction may help with better integrating this content into the manuscript.

**Answer and proposed modifications:**

Thank you for this suggestion. It is indeed much clearer to specify in the introduction that MFT improves parental reflexive function and mentalization. This clarifies the part of the discussion concerning the mentalization results: how the MULTI FAST program enables parents to better understand their teenager's feelings and behaviors.

**Modifications included in the revised text:**

Introduction

Multifamily therapy *(end of paragraph)*

Finally, MFT also enables to work on mentalization with members of the family. Mentalization, as described by Fonagy, is defined as a better understanding of the other person, their mental states, and behaviors (Fonagy et al., 1991). Putting oneself in the shoes of the person one is interacting with enables a person to better understand the meaning of the behavior, emotions, and speech of others. Many exercises in MFT aim to improve mentalization and, in particular, to enhance the parental reflexive function (Fonagy et al., 1991).

*References*

Fonagy P, Steele M, Moran G, Steele H, Higgitt A. The Capacity for Understanding Mental States: the Reflective Self in Parent and Child and its Significance for Security of Attachment. Infant Ment Health J. 1991;13:200‑17

21. While of interest, whether teenagers have returned to school seemed beyond the scope of the study and more appropriate for an efficacy study.

**Answer and proposed modifications:**

We have removed the section on what adolescents do after the MFT program and whether they have returned to school, as this will be the subject of a future efficacy study.

**Modifications included in the revised text:**

~~Have the teenagers gone back to school?~~

~~During the interview with parents six months after the end of MFT, the parents were asked about their teenager and whether he or she had returned to school. This same question was asked in a telephone call twelve months after the end of MFT. Of the 27 adolescents who participated in MULTI FAST groups in 2022 and 2023. 15 had returned to regular school, 5 more had returned to a school with a small class size and continued psychological care, 3 had started training to learn a trade, and 4 had neither returned to school nor started a professional project~~

22. Overall, I thought that the discussion could be more concise, focused on the study findings and referenced to the literature.

**Answer and proposed modifications:**

We've shortened the discussion to make it more concise and more relevant to the results.

We hope you'll find the new discussion more relevant.

23. I don’t see the absence of the adolescent interview data as a limitation; this is another study. Limitations need more consideration.

**Answer and proposed modifications:**

Thank you for your feedback. We had indeed described the perspectives (future research concerning the analyses of the adolescents' interviews and study of the siblings' point of view) but we had not properly thought through our limitations.

**Modifications included in the revised text:**

Because we chose a purely qualitative method, we cannot demonstrate the therapeutic benefit of MFT for adolescents with ABSR and their parents. For example, the presence of other factors, such as treatment intensification, may play a role in the experience of the parents we interviewed. This study thus successfully explores parents' experiences of MFT, but it cannot quantitatively demonstrate its therapeutic benefit in ABSR in adolescents. The relief described by the parents may also be linked to the fact that they were able to access treatment in an adolescent psychiatry department. While France currently has a very high demand for care for adolescents with mental health problems, it has very few adolescent psychiatric departments. In the future, we plan to set up a quantitative study to assess the effectiveness of MFT for adolescents with ABSR and their parents. This quantitative study will complement the qualitative approach presented in this paper.

The results of the analysis of the interviews with the adolescents will be published soon, but the initial analyses confirm the value of MFT from the adolescents' perspective as well as their parents'. The point of view of siblings is also very important, and their presence is fundamental in MFT groups. Research specifically focused on siblings would thus be useful.

24. Minor Comments.
    Line 18. Expression/word choice. I was unclear what an “essential prognostic factor” was.
    Line 23. “Participation in the study was offered…” rather than “The study was offered…”
    Line 32. Tense – “was” rather than “is” as per the prior sentence.
    Line 152. Awkward expression.
    Line 278. Does a “favorable opinion” mean approval? If the ethics committee/IRB could be translated that would improve clarity.
    Line 399-400. I had trouble following the meaning of this quote. Please review.
    Line 418. “appropriateness” might be more suitable than “correctness”. I had trouble following the second half of this sentence beginning with “as well as…”.
    At times the written expression seemed colloquial rather than scientific see line 484.

**Answer and proposed modifications:**

We have corrected the sentences.

**Modifications included in the revised text:**

Line 18: Family and especially parental involvement is essential. ~~is an essential prognostic factor~~

Line 23: Participation in the study was offered ~~The study was offered~~ to all families who participated in a MULTI FAST group

Line 32: The third theme was ~~is~~ the expression of parental distress to their adolescents

Line 145: MFT developed all around the world ~~subsequently~~ ~~developed as well in Europe (Great Britain, Germany, Switzerland), South America, and elsewhere~~, around multiple issues and disorders (35).

Line 317: The ethics committee approved the study on May 9, 2022. ~~The Comité de Protection des Personnes Sud-Est VI gave a favorable opinion on May 9, 2022~~ (Approval Number: 21.04640.000059)

Line 450*: Mother 4: "... he also saw that other moms, other parents, could ask themselves the same questions~~, that it was legitimate~~ ... I think that helped”*

Line 468: Parents nonetheless expressed questions and doubts as to the ~~correctness~~ appropriateness of parental "withdrawal". They wonder whether their adolescent's reactions are related to adolescence or anxiety, ~~as well as the part attributable to the process of adolescence and to anxiety about school~~(Q6).

Line 484: we removed the sentence: “So we must care for parents too!”
