# Supplementary material for: Does multifamily therapy help parents of adolescents with anxiety-based school refusal? A qualitative approach
Source: PLoS One. 2025 Oct 8;20(10):e0334037. doi: 10.1371/journal.pone.0334037 (PMC12507313; doi:10.1371/journal.pone.0334037)
Supplement: Attachment — Submitted filename: Response to reviewers 2.docx [file pone.0334037.s005.docx]

**Title:** **Does multifamily therapy help parents of adolescents with anxiety-based school refusal? A qualitative approach**

**Manuscript N° PONE-D-24-44513**

*Answers to academic editor and reviewers’ comments with modifications we would like to make to the text.*

*The proposed modifications are highlighted in yellow in this document and in the revised version of the manuscript.*

**Academic Editor**

We would like to thank Dr Nadolu for his invaluable advice, particularly on the presentation of the results of the qualitative analysis.

1. One of the first reviewers recommended that you include 2 bibliographic references (MDPI), if they do not fit with your approach please remove them, it is an inappropriate practice for promoting his/her own work.

**Answer and proposed modifications:**

You note that one of the first reviewers recommended that we include two bibliographic references. We were able to use these references in the discussion to articulate them with the objectives worked on during the multifamily therapy sessions, notably to work on family communication, family members' ability to collaborate and parents' expression of positive emotions toward their adolescent. We therefore decided not to remove them.

2. The main observation of the negative review (to which I also subscribe) is that the qualitative analysis is presented too superficially. If you used a coding system, you can, for example, present table 1 in more detail, not only with themes and subthemes but also with their extension (number of words and weight in total interviews) or even a conceptual map of how these are connected.

**Answer and proposed modifications:**

The main observation concerns the presentation of the qualitative analysis results. We coded the transcripts according to the principles of thematic analysis, a qualitative approach involving the active construction of overarching patterns and meaning across a dataset. Thematic analysis allows the flexible and nontheoretical exploration of rich text data to construct themes that reframe, reinterpret, and/or connect elements of the data without developing a final theory. We applied interpretative phenomenological analysis (IPA) to analyze, interpret, and conceptualize our data. This method focuses on how participants make sense of their experiences and does not lend itself to the presentation of the number of words and weight of themes and subthemes in total interviews. On the other hand, your suggestion that we present the results in the form of a conceptual map seems to us to be a truly excellent suggestion, and we are very grateful to you for it. We have therefore added a figure representing the conceptual map, showing the connections between the themes and subthemes. We hope that this more precise and in-depth presentation better meets your expectations and those of PLos One.

**Modifications included in the revised text:**

Iterative thematic analysis enabled us to develop a conceptual map (Figure 1) comprising four domains: (1) Changes in parents’ representations of their adolescent; (2) Parents looking at themselves in a new light; (3) Parents expressing their distress to their adolescent; (4) New interactions. We go on to describe each domain in the four sections that follow. Each section is organized in the same way: the definition of the domain; its division into underlying themes and subthemes; and support of these constructs through representative quotations.

Figure 1: Concept map: domains and themes toward MFT for ABSR: parents’ perspective

3. also, the hermeneutic interpretation of the results should be a little more elaborate, in principle the extracts from the recorded texts used as examples are not longer than the interpretation itself.

**Answer and proposed modifications:**

We agree that in our previous version, the quotations were too numerous and too long, and the hermeneutic interpretation of the results was sometimes too succinct. We have modified the results section to give more detail to the interpretation and rebalance the interpretation/quotation ratio and hope that this reorganization of the results section better meets your expectations.

**Reviewer #2**

After reviewing the revisions to the manuscript "Does Multifamily Therapy Help Parents of Adolescents with Anxiety-Based School Refusal? A Qualitative Approach," I found that the authors have implemented all the required modifications. In its current form, I believe the manuscript is suitable for publication in your journal.

**Reviewer #3**

We would like to thank the reviewer for these comments. Your first comments helped us a lot to improve our manuscript and we hope that these new modifications will make it even better.

1. Thank you for the opportunity to re-review this manuscript. I believe the authors have made improvements; however, some issues remain.
   Line 136-137. This newly added single sentence paragraph is not well integrated.

**Answer and proposed modifications:**

Thank you for your comment. We have better integrated the sentence in the previous paragraph on the role of families in the care offered to adolescents with school refusal.

**Modifications included in the revised text:**

Gonzalvez stresses the importance of working on family functioning to help adolescents with this form of school refusal (15). Maynard et al. reviewed 8 studies describing different treatments for school refusal among children and adolescents; 6 of them actively involved parents (27). These articles show the essential role that families play in the treatment of adolescents with school refusal.

2. Line 312. There can be only one primary diagnosis so, I suggest deleting “at least one” and replace it with “a”.

**Answer and proposed modifications:**

Indeed there can be only one primary diagnosis, thank you for this correction.

**Modifications included in the revised text:**

adolescent aged between 12 and 18 years, whose psychiatrist determined that they met Berg's criteria for school refusal (7) and had a DSM-5 anxiety disorder as primary diagnosis.

3. Line 198-203. As suggested mentalization has been mentioned in the introduction, however it does not seem to be well integrated into the introduction.

**Answer and proposed modifications:**

We agree that we should have integrated mentalization better into the introduction as it is a key MFT concept, and we hope this revision succeeds in doing so. In the introduction, we describe the principal aspects of MFT and show that improving mentalization, together with the reflective function, is a key element of the program and leads to more efficient helping strategies.

**Modifications included in the revised text:**

It enables work with the family: psychoeducation, reducing conflict, improving communication, setting up strategies for returning to school or resuming a project. In MFT, to reduce conflict and improve communication, we work on mentalization within families. Mentalization, as described by Fonagy, enables a better understanding of others, their mental states, and behaviors (44). Putting oneself in the shoes of the person with whom one is interacting makes it possible to apprehend and appreciate the meaning of his or her behavior, emotions, and speech. Many exercises in MFT aim to improve mentalization and, in particular, to enhance the parental reflective function (44), for the recognition of the other's thoughts and feelings improves family communication.

4. Line 313-314 – the period of school absenteeism ranging from 2 consecutive weeks through 18 months sounds like it was a finding rather than a selection criterion. Is that right? If a child had attended one day in the past two weeks or missed 19 months of school, would they be excluded?

**Answer and proposed modifications:**

The period of school absenteeism ranging from 2 consecutive weeks through 18 months is a selection criterion: if an adolescent attended school one day in the past two weeks or missed 19 months of school, he or she would be excluded. In our population, duration of school refusal was 5 to 16 months and the minimum number of school days missed in our study was 92 days.

5. Line 408-420. I think the presentation of the participant characteristics using descriptive statistics is an improvement, however suggest that this information either be presented in a table or in prose rather than dot points.

**Answer and proposed modifications:**

We changed the presentation of the participant characteristics. This information is now presented in prose.

**Modifications included in the revised text:**

*Participants’* *characteristics*

Of the 31 parents, 19 were mothers and 12 fathers. Parents’ages ranged from 41 to 65 years. The mean age was 51.5 (SD= 6.45) for fathers and 48.7 (SD=4.77) for mothers. The country of birth was France for 71% of parents; 19% of parents were divorced and 6% single or not living with a partner. Among the 31 parent participants, 5 (16.5%) were teachers and 5 more hourly workers, 4 (13%) unemployed and 4 others sales workers, 3 (10%) artists and 3 business owners, 2 (6%) engineers and 2 nurses, one retiree, one doctor, and one civil servant. Adolescents were aged from 13 to 17 years old; 48% were girls and 52% boys. Duration of school refusal was 5 to 16 months. In our population, the minimum number of school days missed was 92 days.

6. While the addition of the methodological detail of the data analysis are lengthy, they assist the reader in understanding of the approach.
7. In my view, the lack of description of the themes remains a limitation.

**Answer and proposed modifications:**

Following the advice of academic editor Dr Bogdan Nadolu and your advice, we have modified the presentation of the results to better detail the codes found in the thematic analysis. We have made the description of the themes more detailed and we have therefore added a figure representing the conceptual map, which shows how themes and subthemes are connected. We hope you find that these changes have improved our manuscript.
